# Targeted Protein Degradation Strategies in DNA Virus Research

**DOI:** 10.3390/v18060658

**Published:** 2026-06-09

**Authors:** Michael Lam, Chayah Hill, Ethan Thornburg, Marsha DeSmet

**Affiliations:** Tom and Julie Wood College of Osteopathic Medicine, Marian University, Indianapolis, IN 46222, USA; mlam301@marian.edu (M.L.); chill449@marian.edu (C.H.); ethornburg997@marian.edu (E.T.)

**Keywords:** DNA viruses, PROTACs, antiviral, viral lifecycles

## Abstract

DNA viruses rely extensively on host cellular machinery, including replication factors and transcriptional systems, to persist after infection. These mechanisms make studying and targeting DNA viral proteins challenging, as they also play key roles in mammalian processes. Traditional strategies include CRISPR-mediated gene disruption and small interfering RNA (siRNA) to target host proteins. However, Proteolysis Targeting Chimeras (PROTACs) offer a novel strategy by enabling the selective and rapid degradation of specific viral or host proteins involved in the DNA viral lifecycle. PROTACs are heterobifunctional molecules composed of three key components: a ligand that binds the target protein, a chemical linker, and a ligand that recruits an E3 ubiquitin ligase. By simultaneously binding both the target protein and the E3 ligase, PROTACs form a ternary complex. This proximity enables the E3 ligase to ubiquitinate the target protein, marking it for recognition and subsequent degradation by the intracellular proteasome. This approach represents a promising avenue for targeting previously undruggable proteins and improving therapeutic outcomes in virus-associated malignancies. In this perspective, we describe studies that use PROTACs as tools to modulate host proteins to investigate DNA viral processes with temporal control of host protein expression, as well as the use of PROTACs as antivirals to directly target DNA viral proteins. We also provide a detailed chart summarizing known host-targeting PROTACs and their potential applications across different stages of DNA viral lifecycles, highlighting opportunities for future DNA virus research.

## 1. Introduction

Unlike many RNA viruses, most DNA viruses replicate their genomes within the nucleus and rely extensively on host cellular machinery to complete different stages of their lifecycle. Because DNA viruses typically encode relatively small genomes, they possess a limited number of viral proteins capable of carrying out essential processes such as genome replication, transcription, and episome maintenance. As a result, these viruses must use host replication factors, transcriptional machinery, and DNA damage response pathways to support productive and persistent infection [1,2]. DNA viruses have evolved to hijack host factors necessary to support their replication, creating a continual battle for genomic integrity between the virus and the host, a feature that is not always fully appreciated [3]. However, targeting these host proteins experimentally can be challenging, as many of these factors are also essential for normal cellular function.

Targeted protein degradation technology encompasses a broad range of approaches, including PROteolysis TArgeting Chimeras (PROTACs), hydrophobic tagging, N-degron technology, LYTACs, molecular glues, and related systems [4]. However, this Perspective focuses primarily on PROTAC-based approaches, as they currently represent the most widely utilized targeted degradation strategies in DNA virus research. We discuss how targeted protein degradation technologies have been used to study DNA viral processes and how these molecules have emerged as valuable experimental tools for identifying virus–host interactions within infected cells. We will also briefly highlight the potential of PROTAC-based strategies to target viral proteins as a novel antiviral approach.

## 2. Targeted Protein Degradation Technology with PROTACs

Traditional approaches used to study host protein function, such as CRISPR-mediated gene disruption or siRNA knockdown, often require prolonged depletion of host proteins (24–72 h), which complicates interpretation of viral phenotypes. PROTACs and other targeted protein degradation technologies have emerged as powerful strategies to rapidly and selectively degrade specific proteins [5,6,7,8,9,10]. These molecules recruit E3 ubiquitin ligases to induce proteasomal degradation of target proteins, enabling rapid and reversible depletion of host factors. Here we will describe the use of this technology to study DNA virus biology; however these molecules have also been widely explored in a variety of diseases such as cancer, cardiovascular and neurological diseases [11].

The first PROTAC was designed in 2001 [5]. This peptide-based degrader consisted of a phosphopeptide recognized by the SCFβ-TRCP E3 ubiquitin ligase complex linked to a ligand targeting methionine aminopeptidase-2 (MetAP-2). This study provided one of the first proof-of-concept demonstrations that induced proximity between an E3 ligase and a target protein could drive selective protein degradation. Today’s PROTACs are heterobifunctional molecules consisting of three parts: a ligand that binds the target protein, a linker, and a ligand that recruits an E3 ubiquitin ligase. PROTACs are based on the intracellular ubiquitin–proteasome system (UPS) to degrade target proteins of interest through a multi-step process. First, the PROTAC recruits both the target protein and the E3 ubiquitin ligase, forming a ternary complex; second, the target protein is ubiquitinated by the E3 ligase; third, the ubiquitinated protein is specifically recognized and degraded by intracellular proteasomes; and fourth, the degrader is released and can be reused in the cycle [12].

Unlike CRISPR-Cas systems, which function at the genomic level by introducing targeted DNA double-strand breaks or nucleotide modifications that permanently alter gene expression, PROTACs act at the proteomic level. They recruit E3 ubiquitin ligases to induce selective degradation of disease-causing proteins via the ubiquitin–proteasome system. Thus, CRISPR causes permanent genomic changes, whereas PROTACs are temporal, exerting post-translational, reversible, and catalytic effects that allow for repeated degradation of targeted proteins without permanent genomic consequences for the host or virus [13].

PROTACs typically exhibit a rapid onset of activity because their mechanism induces ubiquitination and proteasomal degradation shortly after cellular exposure. These effects are often measurable within hours due to the efficiency of the ubiquitin–proteasome system. In contrast, CRISPR-based therapeutics take more time, as their mechanism requires cellular delivery, transcription, and DNA repair processes before phenotypic changes are observed; the outcome is also permanent. While CRISPR has long-lasting effects on the genome, PROTACs offer similar functional outcomes without permanent genomic alteration. These differences in timing and reversibility are particularly important when considering adverse events. PROTACs may degrade off-target proteins, but this effect is often reversible. In contrast, off-target effects from CRISPR-based therapeutics result in permanent genomic changes [14,15].

**Figure 1:**
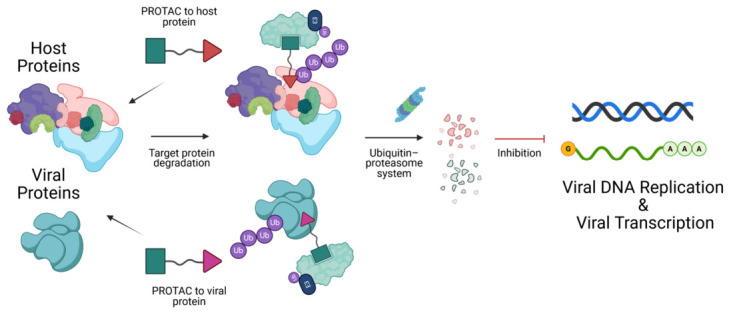
DNA viruses rely extensively on host transcriptional and replication machinery to support viral gene expression, genome replication, and productive infection. PROTACs bind to target proteins (triangle) to recruit E3 ubiquitin ligases (square) to selectively ubiquitinate and degrade host or viral proteins through the ubiquitin–proteasome system. Degradation of proteins from the host (top) or the virus (bottom) required for the viral lifecycle disrupts viral transcription, replication, and other essential processes, highlighting the utility of PROTACs as powerful and reversible tools to study DNA virus biology. Figure created in BioRender. DeSmet, M. (2026) https://BioRender.com/ sh66u3j.

PROTAC technology provides a novel opportunity to study DNA viruses. PROTACs can be used to provide short-term, reversible degradation of host proteins involved in their lifecycles. At a more complex level, they can also be designed to directly target viral proteins (Figure 1). These areas will be discussed in more detail in the following sections.

## 3. Host Factor Degradation Reveals Viral Dependencies

DNA viruses rely on host proteins to complete their lifecycles; therefore manipulation of these host proteins is necessary to identify their functions. Here, we describe the use of PROTACs to modulate host protein activity in viral models. These tools provide unique capabilities to tightly control host protein expression.

Hepatitis B virus (HBV) is a partially double-stranded relaxed circular DNA virus that infects hepatocytes and is associated with both acute and chronic liver disease. Chronic infection can lead to hepatocellular carcinoma (HCC). The HBV genome is converted into covalently closed circular DNA (cccDNA), a chromatinized episomal genome that serves as the master template for all HBV transcription. Because cccDNA is organized like host chromatin, wrapped around histones and regulated by epigenetic machinery, Yu et al. [16] reasoned that the virus must be actively co-opting host transcriptional regulators to drive its own gene expression. Through a screen of 146 compounds, they identified BRD4, a host protein that normally promotes gene activation, as a critical factor that HBV utilizes to sustain cccDNA transcription. In this study, BRD4 PROTACs were used as a validation tool. Depleting BRD4 with PROTAC degraders (dBET1 and MZ-1) suppressed viral RNA production in infected cells, supporting that HBV transcription depends on BRD4. Both JQ1 (inhibitor) and PROTACs suppress HBV transcription, but PROTAC-mediated degradation produces a more complete loss of BRD4 function and results in a more pronounced reduction in viral RNA. This suggests that BRD4 contributes to HBV transcription through mechanisms that are not fully inhibited by blocking bromodomain-mediated interactions alone [16]. This distinction illustrates the unique power of targeted degradation as a research tool: by eliminating the protein rather than just blocking its activity, PROTACs can reveal functional dependencies that conventional inhibitors mask.

Human cytomegalovirus (HCMV) is a double-stranded DNA virus of the herpesvirus family that establishes lifelong infection. The virus relies on host cyclin-dependent kinases (CDKs), including CDK9, to promote viral gene expression and replication. Hahn et al. [17] treated HCMV-infected foreskin fibroblasts (HFFs) with the CDK9 PROTAC, THAL-SNS032. CDK9 depletion significantly reduced HCMV replication. Interestingly, treatment of these infected cells with the CDK9 inhibitor SNS032 also reduced HCMV replication, but not to the same extent as removing CDK9 using the PROTAC derived from SNS032. The PROTAC demonstrated greater antiviral activity, highlighting the importance of host protein removal compared to host protein inhibition.

The human papillomavirus (HPV) is a double-stranded DNA tumor virus that relies on host machinery to maintain its genome as an episome during replication and for viral transcription. However, studying these host proteins in the context of viral infection is challenging due to their essential roles in normal cellular processes. To address this, PROTACs have been utilized to enable precise control of host protein expression. For example, BRD4 has been identified as essential to the different steps in the HPV lifecycle [18,19]. Treatment of HPV-31 episomal cells (CIN612) with a BRD4 PROTAC demonstrated the importance of this protein during early viral transcription [20]. In a separate study, Jose et al. [21] treated keratinocytes with a tyrosine kinase focal adhesion kinase (FAK) PROTAC and found that its depletion suppressed HPV replication after infection but not during genome maintenance or amplification. These studies highlight the use of PROTACs to investigate the roles of host proteins across different stages of the HPV lifecycle that are not fully revealed by traditional inhibition strategies.

## 4. Protein Degradation Anti-Viral Targets

PROTACs present a promising strategy to target DNA viral proteins that are traditionally considered undruggable. However, only a limited number of PROTACs targeting these viral proteins have been developed. In the next section, we discuss known DNA viral targets and their impact on inhibiting the viral lifecycle or persistence.

To create a PROTAC targeting the HPV-16 oncogene, E6, a panel of nanobodies were generated against the protein [22]. Nanobody A5 exhibited the highest affinity toward E6 and was fused with Von Hippel-Lindau (VHL) protein to target E3 ligase activity. Results revealed that the A5-VHL fusion bioPROTAC was able to degrade E6 in vitro resulting in restoration of the tumor suppressor protein p53, which is normally targeted for degradation by E6. Mice with established TC-1 tumors (HPV-16 E6 and E7 positive) were injected at Day 7 and Day 14 with the PROTAC demonstrated a reduction in tumor growth by Day 21 [22]. These findings highlight the potential of PROTAC technology to target viral oncogenes.

Kaposi sarcoma-associated herpesvirus (KSHV) is a double-stranded DNA gammaherpesvirus and a major etiological agent of several malignancies, including Kaposi sarcoma (KS) and primary effusion lymphoma (PEL). The latency-associated nuclear antigen (LANA) is a key viral protein that promotes immune evasion and supports tumorigenesis during latent infection. LANA is essential for latent replication by tethering viral episomes to host chromosomes to ensure the genome is replicated once per cell cycle. A BAC16-derived KSHV genome was engineered to fuse the mini auxin-inducible degron system (mAID) to LANA (mAID-LANA) [23]. Upon addition of the synthetic auxin 5-Ph-IAA, the SCF–OsTIR1 E3 ubiquitin ligase complex is recruited to the mAID tag, leading to polyubiquitination and rapid degradation of mAID-LANA in iSLK cells. After 1.5 h of auxin treatment, Western blot analysis demonstrated a significant reduction in mAID-LANA levels, resulting in a decrease in KSHV DNA. The authors used this system as a tool to manipulate LANA levels. Using this inducible degradation system, Nakajima et al. found that in cells lacking cGAS, a key sensor of the innate immune system, KSHV DNA was not degraded in the presence of auxin. These results suggest that cGAS is necessary to detect the KSHV genome.

A complementary approach utilized HaloTag-fused LANA targeted by HaloPROTAC3 [24]. In this system, LANA was fused at its N-terminus to a HaloTag to enable targeted degradation using HaloPROTAC3. HaloPROTAC3 binds covalently to the HaloTag to recruit the VHL E3 ubiquitin ligase complex, resulting in ubiquitination and degradation of LANA [23,25]. HaloTag–LANA encoded within the recombinant KSHV genome was efficiently degraded in iSLK.BAC16 cells following treatment with HaloPROTAC3. Consistent with this, depletion of LANA resulted in a significant reduction in KSHV episome maintenance within 48 h. Although these studies demonstrate proof-of-principle for targeted degradation of LANA, it does not represent a directly translatable therapeutic strategy for KSHV infection, as it relies on engineered tagged LANA fusion proteins rather than the native viral protein.

Epstein–Barr virus (EBV) is a double-stranded DNA virus with oncogenic properties associated with several malignancies, including nasopharyngeal carcinoma (NPC), gastric carcinoma, and multiple subtypes of lymphoma. EBV encodes Epstein–Barr virus nuclear antigen 1 (EBNA1), which interacts with host proteins such as USP7 and the MCM complex, contributing to viral persistence, immune evasion, and tumor progression. A PROTAC, EP-1215, was designed to target the EBNA1 C-terminal DNA-binding domain [26]. EP-1215 induced degradation of EBNA1 in cells and in xenograft models that overexpress EBNA1. Using this PROTAC, Liu et al. [27] demonstrated that EBNA1 upregulates ADAR1, an RNA editing enzyme that converts adenosine to inosine, thereby suppressing host interferon signaling and promoting tumor immune evasion. The EBNA1-targeting PROTAC elucidated the importance of this viral protein in tumor immune suppression. These findings highlight the dual utility of PROTACs as both therapeutic strategies and tools to uncover the multifaceted roles of viral proteins.

HBV encodes a regulatory protein, the X protein (HBx), which activates viral transcription from cccDNA and contributes to carcinogenesis. Montrose et al. [28] designed a non-traditional PROTAC-like construct by combining a DDB1-derived HBx-binding domain with an oligomerization domain, enabling multivalent binding of HBx and recruitment of the CUL4–DDB1 E3 ligase complex to promote its degradation. In hepatic cells that overexpressed HBx, treatment with a non-traditional, peptide-based PROTAC-like construct induced HBx degradation. However, the impact of this degradation on the HBV lifecycle was not determined.

**Figure 2:**
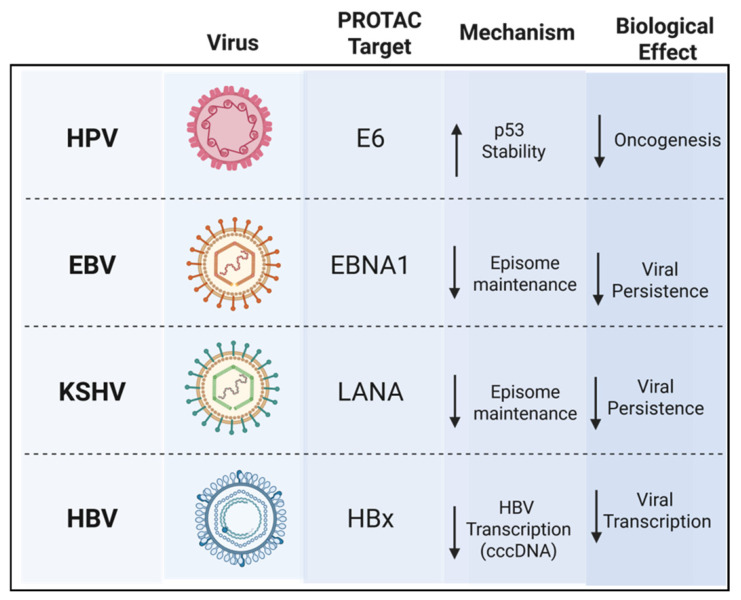
PROTAC-mediated degradation of viral proteins disrupts key mechanisms of DNA virus persistence, transcription, and oncogenesis. Listed are published PROTACs generated targeting viral proteins and their associated mechanisms and biological effects. Figure made with Created in BioRender. DeSmet, M. (2026) https://BioRender.com/ nr888mf.

In conclusion, PROTACs offer a powerful strategy for targeted protein degradation, enabling the elimination of protein function and offering therapeutic potential against proteins implicated in viral persistence. These studies demonstrated that PROTACs not only promote degradation of viral proteins (e.g., EBNA1, HBx, E6, and LANA) but also disrupt their roles in viral replication and tumor progression (Figure 2). This is exemplified by the A5-VHL bioPROTAC, which effectively degraded E6 in mice, leading to a reduction in tumor growth. However, the use of PROTACs as antivirals is hindered by several limitations, including reliance on engineered tagging systems, the need for ligands with high affinity and precise target selectivity, and challenges in achieving efficient cellular delivery. In addition, the HPV oncoproteins such as E6 and E7 are intracellular and nonenzymatic, lacking well defined binding pockets, which complicates effective PROTAC-based strategies. Furthermore, the relatively large molecular size of PROTACs restricts the pharmacological and delivery performance of these molecules in vivo. Overall, despite significant challenges, continued advances in targeting strategies and ligand design may enable PROTACs to emerge as future therapeutic antivirals.

## 5. Future Targets with Known PROTACs to Study DNA Lifecycles

Studying the lifecycles of DNA viruses is particularly challenging because these viruses are highly dependent on host cellular machinery for replication. As a result, it is often difficult to distinguish virus-specific functions from host-driven processes. In addition, modeling latency and reactivation remains a significant challenge, as these states are dynamic and tightly regulated by host factors. The temporal complexity of DNA viral lifecycles, combined with their reliance on host proteins, further complicates experimental design and their interpretation. There are limited tools available for precise manipulation of DNA viral genomes, particularly in the context of manipulation of the host proteins necessary to carry out their lifecycles.

The development of PROTACs presents an exciting opportunity to overcome some of these challenges. As described in this Perspective, researchers are increasingly utilizing this technology to selectively target and degrade viral and host proteins involved during the infection and persistence of DNA viruses. Traditional inhibitors and small-molecule drugs typically provide only transient suppression of protein inhibition and are often limited to enzymatic targets. In contrast, PROTACs enable sustained suppression of protein function through targeted degradation. In addition, PROTACs often require lower doses and may reduce the likelihood of resistance.

Several host proteins with established roles in DNA viral lifecycles are now considered PROTAC-accessible targets. These proteins, along with their functions in viral infection, are summarized in Table 1. As the field continues to expand, an increasing number of PROTACs are being developed to target pathways critical for DNA virus replication, persistence, and transcriptional regulation. As shown in Table 1, DNA damage response proteins (e.g., ATM/ATR, CHK1, WEE1) are commonly exploited across multiple DNA viruses, including HPV, KSHV, EBV, HBV, adenovirus, HSV, and polyomavirus, where they facilitate viral genome replication and maintenance. In addition, chromatin and transcriptional regulators (e.g., BRG1, BRD4, CDK9) exhibit broad functional overlap across DNA viruses, supporting processes such as viral transcription, episome maintenance, and oncogene expression. Another area of focus has been the broad targeting of RNA and DNA viruses through host-directed degradation strategies. For example, FM-74-103 induces degradation of human GSPT1, a translation termination factor shown to be required for influenza A virus, SARS-CoV-2, and cytomegalovirus (CMV) replication [29]. Table 1 summarizes host proteins with available PROTAC degraders that have established roles in DNA virus lifecycles and may represent useful tools for future mechanistic studies. Together, these observations highlight a convergence of DNA viruses on host pathways that regulate genome stability and transcriptional control.

## Figures and Tables

**Table 1:** Host proteins with available PROTAC degraders that have established roles in DNA virus lifecycles.

PROTAC- Targetable Host Protein	DNA Viruses Associated with the Host Protein	Role in Viral Lifecycle
CDK9 [30] Transcription regulator	CMV [17] HPV [31] HBV [32]	CMV: promotes viral transcription elongation via RNA polymerase II phosphorylation HPV: supports viral gene transcription through RNA polymerase II activation HBV: regulates transcription from HBV cccDNA
ATM/ATR [33,34] DNA damage response	HPV [2,35] KSHV [36,37] EBV [38] HBV [39] Adenovirus [40] HSV [41] Polyomavirus [41]	HPV: activates DNA damage signaling required for viral genome replication KSHV: DDR signaling supports lytic viral DNA replication EBV: DDR signaling facilitates viral replication and persistence HBV: HBx activates ATR-dependent pathways linked to replication and carcinogenesis Adenovirus: suppresses ATR signaling to permit viral replication HSV: required for efficient lytic replication Polyomavirus: supports viral DNA replication and genome maintenance
WEE1 [42] DNA damage response	HPV [43]	HPV: regulates the G2/M checkpoint exploited for viral genome amplification
EZH2 [44] Chromatin regulation	EBV [45,46]	EBV: H3K27 methylation regulates viral latency and gene expression
BRG1-SWI/SNF [47,48] Chromatin regulation	HBV [49,50] HPV [51]	HBV: chromatin remodeling promotes transcription from viral cccDNA HPV: supports oncogene transcription and E2-dependent replication
FGFR1/2 [52,53,54] Receptor signaling	HPV [55]	HPV: FGFR signaling regulates E2 activity and viral transcription
BRD4 [56] Transcription regulator	HPV [18,19] Polyomavirus [57] EBV [58,59] HBV [60] KSHV [61]	HPV: regulates E2-dependent transcription, replication, and genome tethering Polyomavirus: promotes viral DNA replication via large T antigen interaction EBV: supports transcription through EBNA1 and pTEFb interactions HBV: regulates transcription from viral cccDNA KSHV: mediates episome segregation through LANA interaction
CHK1 [53] DNA damage response/checkpoint kinase	Polyomavirus (BKPyV) [62] Adenovirus [63] Parvovirus [64]	Polyomavirus: regulates cell cycle checkpoints exploited for viral DNA replication Adenovirus: ATR-CHK1 signaling restricts viral replication Parvovirus: restricts viral replication; virus inhibits CHK1 to enhance replication
PARP1 [65] DNA repair and chromatin regulation	HBV [66] EBV [67] KSHV [67]	HBV: promotes viral replication via core promoter interaction; virus uses PARP1 to suppress its own ADP-ribosylation EBV: regulates latency and gene expression; virus modulates PARP1 for replication KSHV: facilitates episome maintenance and lytic replication; virus regulates PARP1 to evade restriction
CypA (PPIA) [68] Peptidyl-prolyl isomerase chaperone	HBV [69]	HBV: facilitates viral replication and HBsAg secretion
GSPT1 [29] Translation termination factor	CMV [29]	CMV: crucial host factor for viral replication; degradation inhibits infection
